# The Relevance of β-Thalassemia Heterozygosity in Pediatric Clinical Practice: Croatian Experience

**DOI:** 10.3390/children11070785

**Published:** 2024-06-27

**Authors:** Ana Dordevic, Milena Ugrin, Ines Mrakovcic Sutic, Jelena Roganovic, Sonja Pavlovic

**Affiliations:** 1 Jadran Galenski Laboratorij, 51000 Rijeka, Croatia; ana.dordevic@jglpharma.com; 2 Institute of Molecular Genetics and Genetic Engineering, University of Belgrade, 11000 Belgrade, Serbia; milena.ugrin@imgge.bg.ac.rs (M.U.); sonya@imgge.bg.ac.rs (S.P.); 3 Faculty of Medicine, University of Rijeka, 51000 Rijeka, Croatia; ines.mrakovcic.sutic@medri.uniri.hr; 4 Children’s Hospital Zagreb, 10000 Zagreb, Croatia; 5 Faculty of Biotechnology and Drug Development, University of Rijeka, 51000 Rijeka, Croatia

**Keywords:** beta-thalassemia, genotype, screening, Croatia, pediatric

## Abstract

(1) Background: Thalassemia syndromes are common monogenic disorders that represent a significant global health issue. No systematic epidemiological or molecular investigations on thalassemias in the Croatian population have been reported to date. (2) Methods: This prospective study included 70 children with a presumptive diagnosis of thalassemia and their 42 first-degree relatives. Molecular characterization was performed using direct sequencing and gap-PCR methods. (3) Results: We identified 46 (30 children and 16 first-degree relatives) β-thalassemia heterozygous carriers from 24 unrelated families, carrying eight different mutations and one hemoglobin variant. Five variants account for approximately 85% of all affected β-globin alleles: Hb Lepore-Boston-Washington (32.6%), HBB:c.93-21G>A (19.6%), HBB:c.315+1G>A (13.1%), HBB:c.92+1G>A (10.9%), and HBB:c.92+6T>C (8.7%) variants. (4) Conclusions: β-thalassemia carriers need more detailed genetic profiling since genetic modifiers can significantly impact their phenotype. Our study provides important new insights into the relevance of β-thalassemia heterozygosity in pediatric clinical practice.

## 1. Introduction

Beta-thalassemias are a heterogenous group of inherited blood disorders that result from the defective synthesis of β-globin chains leading to ineffective erythropoiesis and hemolysis. Beta-thalassemias include two main categories based on transfusion requirements: transfusion-dependent thalassemia (TDT), which includes severe forms such as thalassemia major (also known as Cooley anemia or Mediterranean anemia), and non-transfusion-dependent thalassemia (NTDT), which includes milder forms such as thalassemia intermedia and thalassemia minor (also known as heterozygous β-thalassemia or β-thalassemia trait) [1]. The severity of the disease is determined mainly by the extent to which the synthesis of β-globin chains of hemoglobin (Hb) is reduced (β+ and β++) or absent (β0), resulting from the underlying molecular defects on β-globin (*HBB*) gene located on chromosome 11. The clinical spectrum is broad, ranging from asymptomatic individuals with mild microcytic hypochromic anemia to severe TDT. The remarkable phenotypic variability is primarily due to striking variations in the *HBB* gene, with over 300 variants reported [2].

Beta-thalassemia is one of the most common monogenic disorders worldwide, with approximately 1.5% of the global population (80–90 million people) being carriers and 60,000 symptomatic newborns annually [1,3,4]. It is more prevalent in tropical and subtropical areas, extending from sub-Saharan Africa, the Mediterranean, the Middle East, and Southeast Asia (“thalassemia belt”) due to the presence of malaria [5].

Croatia is located at the crossroads of Central Europe, the Balkans, and the Mediterranean [6]. Although at the edge of the traditional thalassemia belt, neither systemic epidemiological nor genetic studies of thalassemia syndromes in the Croatian population have been performed, and there are only a few case reports and one study with a limited number of patients [7]. The only large-scale population study ever conducted included >35,000 subjects from Balkan countries (former Yugoslavia). The frequency of heterozygous thalassemia was 0.8% in Croatia (before the country declared its independence), 1.5% in Bosnia and Herzegovina, 1.8% in Serbia, 1.9% in Montenegro, and 2.9% in North Macedonia (varying from 1.0% to 7.8% in different regions, the highest being close to Greece) [8].

Our study was performed to analyze the spectrum of the β-globin gene variants and to provide baseline data useful in launching the carrier screening, genetic counselling, and prenatal diagnosis of β-thalassemias in Croatia. Additionally, we highlight the importance of the molecular diagnosis of heterozygous β-thalassemia and its implication for carriers’ health.

## 2. Materials and Methods

### 2.1. Subjects

One hundred and twelve subjects (47 females and 65 males) from 65 unrelated families were included in the study. From 1 July 2021 to 30 June 2023, 70 children with microcytic hypochromic anemia, low red blood cell (RBC) indices, and/or elevated levels of HbA2, with a presumptive diagnosis of thalassemia, were recruited from the Clinical Hospital Centre (CHC) Rijeka, Croatia, along with their 42 first-degree relatives with a history of anemia. DNA analysis was performed at the Institute of Molecular Genetics and Genetic Engineering, Belgrade, Serbia. The study was approved by the Ethical Committee of CHC Rijeka (No 2170-29-02/1-21-2; 31 May 2021).

### 2.2. Methods

Hematological parameters (RBC, Hb, mean corpuscular volume [MCV], and mean corpuscular hemoglobin [MCH]) were obtained by an automated hematology analyzer (Sysmex XN-1000; Sysmex Europe GmbH, Norderstedt, Germany). The values were compared with age- and sex (>12 years)-related reference intervals for children [9].

Hb fractions were detected by the capillary zone electrophoresis method using the automated Sebia Capillarys 2 Flex Piercing System (Sebia, Lisses, France). EDTA-whole blood specimens were subjected to onboard hemolysis prior to capillary injection and separated by electrophoretic mobility in an alkaline buffer. The quantitation of eluted fractions was performed spectrophotometrically at 415 nm, and peaks (electrophoregrams) were evaluated visually based on their migration within defined zones. The values were compared with reference intervals of the in-hospital laboratory. In healthy children > 2 years, HbA accounts for more than 96% of the total Hb, HbA2 is 2.5 to 3.5% of Hb, and HbF constitutes less than 1% of total adult Hb [10]. HbA2 ≥ 4.0 % was the cut-off for establishing β-thalassemia carrier diagnosis.

Genomic DNA was obtained from peripheral blood (3 mL) collected in the sodium citrate tubes. The polymerase chain reaction (PCR) used to amplify the *HBB* gene was performed in a total volume of 30 μL, and the reaction mixture contained 20 pmol of each primer (E1,2F: 5′–ACCTCACCCTGTGGAGCCAC–3′/E1,2R: 5′–AATCATTCGTCTGTTTCCCA–3′; E3F: 5′–TCATATTGCTAATAGCAGCTACAATCGAGC–3′/E3R: 5′–CACTGACCTCCCACATTCCC–3′; IVS2F: 5′–AGAACTTCAGGGTGAGTCTATGGG–3′/IVS2R: 5′–TGTGGGAGGAAGATAAGAGGTATG–3′), 120 ng of genomic DNA, 200 μmol/L of each dNTP (Thermo Fisher Scientific Waltham, MA, USA), 1× PCR reaction buffer (Qiagen, Hilden, Germany), 1× Q solution (Qiagen), 2.75 mM MgCl_2_, and 1 U HotStart Taq DNA polymerase (Qiagen). The temperature profile for the initial activation of DNA polymerase was set at 95 °C for 15 min, followed by 35 cycles of 1 min denaturation at 95 °C, 1 min annealing at 57 °C, and 2 min elongation at 72 °C, followed by a final extension period of 10 min at 72 °C.

The PCR products were purified with a QIAquick PCR purification kit (Qiagen Inc.) and used for the direct PCR product sequencing in both directions with a BigDye terminator kit (Applied Biosystems, Carlsbad, CA, USA), using the SeqStudio Genetic Analyzer (Thermo Fisher Scientific) according to the manufacturer’s instructions with the same primers used for PCR amplification. The detection of the Hb variant Lepore-Boston-Washington (Hb Lepore-BW) was performed by gap-PCR analysis as previously described [11].

### 2.3. Statistical analysis

Categorical variables are presented as absolute numbers and frequencies. Continuous variables, presented as medians or means with SEM (standard error of the mean), were analyzed by Student’s *t*-test. The Shapiro–Wilk test was used to assess the data distribution.

Statistical analysis was performed using the SPSS 21.0 software (IBM). For all analyses, *p*-values were 2-tailed, and the significance was *p* < 0.05.

## 3. Results

The mean age of the 70 children included in the study was 9.9 years (range 0.4–17.1 years).

### 3.1. Genotype Analysis

Out of 112 cases belonging to 65 unrelated families, we identified eight different thalassemia mutations and one Hb variant in a total of 46 cases (30 children [mean age 8.9 years; range 0.4–17.1 years] and 16 first-degree relatives; 21 females and 25 males) from 24 unrelated families. All variants were detected in the heterozygous form.

Hb Lepore-BW was the most common cause of thalassemia in our cohort, with a frequency of 32.6%. More than 19% of subjects carried the HBB:c.93-21G>A variant, and 13.1% carried the HBB:c.315+1G>A variant. The third most frequent variant was HBB:c.92+1G>A (10.9%), followed by HBB:c.92+6T>C (8.7%). Altogether, these five variants accounted for up to 85% of all affected β-globin alleles. Two rare β-globin gene variants were also detected—Hb Monroe, with a frequency of 4.3%, and polyA (A>G), with a frequency of 2.2%, as presented in Table 1. Iron deficiency anemia was excluded in all subjects, while the analysis of α-globin gene variants was not performed.

### 3.2. Hematological Parameters and Hb Levels of β-Thalassemia Carriers

As expected, hematological and biochemical parameters of all heterozygote carriers were consistent with the β-thalassemia phenotype, as described in Table 2 and Table 3. Overall, their MCV and MCH values were lower than age- and sex-matched healthy controls, while HbA2 and HbF levels were slightly elevated. Carriers of Hb Lepore-BW had significantly higher MCH values (mean = 20.283 ± 0.400 pg) compared to carriers of other *HBB* variants (mean = 18.578 ± 0.519 pg; *p* = 0.034, *t*-test). On the other hand, levels of HbA2 were lower in Hb Lepore-BW carriers (mean = 2.409 ± 0.0939%) compared to carriers of other *HBB* variants (mean = 4.841 ± 0.136%; *p* < 0.001, *t*-test). In addition, we observed a trend toward a higher mean MCV value in Hb Lepore-BW carriers (mean = 61.05 ± 1.276 fl) in comparison to carriers of other β-globin gene variants (mean = 57.011 ± 1.631 fl; *p* = 0.098, *t*-test).

### 3.3. β-Thalassemia Carriers in Clinical Practice

Twenty-two out of 30 (73.3%) children with the confirmed β-thalassemia trait and no iron deficiency received previous oral iron therapy with a daily dose of 3–5 mg/kg elemental iron for a period of 3 weeks to 4 months, after which the primary physician repeated the complete blood count. As no improvement in Hb and RBC indices was observed, children were referred to the pediatric hematologist for further evaluation, where they received advice to discontinue oral iron. The carriers/their parents were also educated about the importance of molecular testing on genes affecting the metabolism of bilirubin, iron, and bone, such as *UGT1A1* variants, *HFE* variants, *VDR*, *COLIAI*, *COLIA2*, and *TGFB1* variants, to predict possible secondary β-thalassemia trait complications.

## 4. Discussion

Beta-thalassemias are a heterogeneous group of inherited Hb disorders characterized by reduced or absent β-globin chain synthesis. Historically, thalassemias have been most frequent in subtropical malaria-endemic regions of the world, reflecting the relative resistance of carriers to *Plasmodium falciparum* and the higher frequency of consanguineous marriages [1,12]. Due to large-scale migrations, the prevalence of β-thalassemia is continuously increasing in non-endemic regions, including Northern and Western Europe and North America, and making this disease a global health concern [5].

A limited number of studies have reported population-based estimates of β-thalassemia, ranging from 0.2/100 000 people in Spain in the period 2014–2017 to 49.6/100 000 people in Iraq in the period 2003–2018 [13,14], and varying even within countries [15]. To better understand the global β-thalassemia burden and to help direct public health policies, up-to-date epidemiological data are needed for many countries. Disease-causing variants in thalassemias are often population-specific. There is a particular paucity of data for Croatia. Our study presents the most extensive national study to date, comprising 46 β-thalassemia cases originating from Croatian Littoral and Istria, and could help to formulate a Croatian carrier screening program. Currently, effective programs, including guidelines for hematological and molecular methods for carrier identification, recommendations for genetic counselling, and referral for prenatal diagnosis do not exist.

The molecular basis of β-thalassemias has been studied in many countries. Only 20 variants account for more than 80% of the β-thalassemia variants worldwide due to geographical clustering. Although the incidence and the prevalence of thalassemia are similar in Croatia compared to its neighboring countries (up to 3% and up to 15%, respectively) [16], each population has a few common variants and a varying number of rare ones [17]. Hb Lepore-BW is the predominant cause of β-thalassemia in Croatia, with a frequency of 32.6%. The results are similar to those of our neighboring country Serbia, with a reported incidence of Hb Lepore-BW of 26.2% [18]. This structurally abnormal form of Hb is the result of the fusion of the β-globin (*HBB*) and δ-globin (*HBD*) genes, and our results demonstrated significantly lower levels of HbA2 (δ_2_α_2_) in the Hb Lepore-BW group compared to carriers of other *HBB* variants. These findings correspond to the previous studies and could be explained by a decreased synthesis of α-globin chain [19,20].

The most common mutations affecting the *HBB* gene in our cohort, HBB:c.93-21G>A (β^+^ IVS-I-110), HBB:c.315+1G>A (β^0^ IVS-II-1), HBB:c.92+1G>A (β^0^ IVS-I-1), and HBB:c.92+6T>C (β^+^ IVS-I-6), were detected in more than 52% of all affected β-globin alleles. These results are very similar to the results of other European countries (Romania, Greece, Bulgaria, Hungary, North Macedonia, and Italy). However, although the frequency of the HBB:c.118C>T (codon 39) variant was relatively high in the neighboring countries (Italy 44.8%; Hungary 29.4%; Bulgaria 29.1%; Greece 19.51; Serbia 16.2%; Romania 16.0%), our results showed an incidence of only 4.3%, probably due to the genetic drift [21,22,23,24,25,26].

Five *HBB* gene variants account for approximately 85% of all β-thalassemia variants in the Croatian population. These findings are in accordance with the observation that each population has a few common variants [27], enabling the choice for the population-specific targeted carrier screening methods. Although thalassemia is sporadic in Croatia, the results might provide information on the history and origin of the different β-thalassemia variants. The common history and possible common ancestry can explain a similar high frequency of Hb Lepore-BW in Croatia and Serbia. The second most frequent IVSI-110 variant, previously reported to be of Eastern Mediterranean (Turkish) origin, probably reflects historical migrations over the Balkan peninsula [7]. The overall similarity of the five most expected Croatian *HBB* gene variants to those reported in other European countries can be attributed to Croatia’s territorial proximity and geographic position at the crossroads of Central Europe, the Balkans, and the Mediterranean.

Although DNA testing for thalassemia trait is not a routine procedure, there are several reasons why genetic studies of β-thalassemia heterozygosity are essential.

Unresolved laboratory hematology and implications for pediatric practice. In β-thalassemia minor, a complete blood count usually shows no or mild anemia (Hb > 9–10 g/d), RBC count is increased or normal, and MCV and MCH are decreased. The peripheral blood smear examination reveals microcytosis, hypochromia, and variations in RBC size and shape. The reticulocyte count is normal or slightly elevated [28]. The differential diagnosis of iron deficiency anemia (IDA) is essential, foremost for the avoidance of unnecessary investigations and for treatment planning [29,30]. The RDW (RBC Distribution Width) is elevated in more than 90% of persons with IDA and in only 50% with heterozygous thalassemia [31]. Various discriminative hematological indices have been proposed for IDA and β-thalassemia traits, each with some degree of inaccuracy. In children, the Mentzer index (MCV/RBC) can help distinguish; in IDA, the ratio is usually greater than 13 and in thalassemias less than 13, whereas a ratio of 13 is considered uncertain [31,32].

The diagnostic confirmation of these two entities often requires further tests involving serum ferritin and Hb electrophoresis. The measurement of the serum ferritin level is the most accurate test to diagnose IDA. In the absence of inflammation, a normal ferritin level (>15 ng/mL) generally excludes iron deficiency. Hb electrophoresis in thalassemia carriers usually demonstrates reduced HbA, increased levels of HbA_2_ (>3.5% of total Hb), and increased HbF (>1%). High-performance liquid chromatography (HPLC) and capillary electrophoresis (CE) are two standard techniques used for quantifying HbA_2_. However, a normal concentration of HbA_2_ does not rule out β-thalassemia trait, especially if there is concomitant iron deficiency, which can lower HbA_2_ levels into the normal range. Furthermore, borderline HbA_2_ values may occur due to mild/silent *HBB* mutations and co-inherited β-thalassemia and α- or δ-thalassemia. As conventional techniques may not be reliable, only confirmation with molecular genetic testing provides accurate diagnosis [33,34,35].

Many β-thalassemia carriers are erroneously believed to have IDA. In our study, 22 out of 30 (73.3%) children with the confirmed β-thalassemia trait received previous oral iron therapy with no improvement. It is important to remember that children with thalassemia trait-related anemia should not take iron supplements unless they have a concomitant iron deficiency. However, several studies reported an underestimation of the coexistence of iron deficiency and thalassemia trait in children [36,37,38]. This coexistence should not be neglected, and iron therapy should be administered in iron-deficient children. We propose that if Hb < 11 g/dL in a case of thalassemia minor, one should screen for iron deficiency simultaneously.

Genetic counselling. Severe forms of thalassemia rarely escape from clinical diagnosis. Beta-thalassemia minor is the heterozygous state that is usually asymptomatic and can be easily dismissed. Carriers are frequently unaware of their disorder. As a rule, thalassemia trait is identified during the screening because of an affected family member or rarely incidentally during routine laboratory analysis, e.g., HBA1c values in diabetic patients [39]. Molecular analysis is the only definitive way to diagnose heterozygous thalassemia and can help qualify which *HBB* variant family’s harbor.

Genetic counselling is inseparable from genetic diagnosis, allowing couples at risk to make informed decisions on their reproductive choices. The extreme phenotypic and molecular heterogeneity of β-thalassemias and the potential co-inheritance of various abnormal Hb require an experienced genetic counselor. Simplifying complex information, if one partner is a known carrier and planning to start a family, another partner should be tested as well. Thalassemias are inherited in an autosomal recessive manner. Therefore, through genetic counselling, ideally in the pre-conception period or as early as possible in the pregnancy, and with the possibility of prenatal diagnosis, the birth of a child with thalassemia major can be avoided, if desired.

Genetic testing improves the healthcare of adult β-thalassemia carriers. The timely screening can be performed for the direct benefit of adult carriers. It is well documented that the remarkable phenotypic diversity of β-thalassemia individuals is associated with a great genotype variety. The primary genetic determinants are mostly different types of *HBB* gene mutations (β^0^/β^+^/β^++^) leading to the decreased or absent production of β-globin chains. However, the causal relationship between phenotype and genotype might be further complicated by the interaction of secondary and tertiary genetic modifiers. Two important secondary modifiers—the co-inheritance of α-thalassemia and variants associated with increased HbF synthesis—have emerged, but they do not explain all clinical heterogeneity [40]. The genes involved are *HBA*, *HBG*, *BCL11A*, *HBS1L-MYB*, and other cofactor genes regulating erythropoiesis [41]. Recent studies revealed that other genetic modifiers, not affecting globin imbalance directly, might moderate secondary manifestations of heterozygous β-thalassemia and response to therapies. Among these, one of the best delineated are those affecting the metabolism of bilirubin, iron, and bone. UDP-glucuronosyltransferase (*UGT1A1*) gene variants (Gilbert syndrome) predispose to jaundice and the formation of gallstones. The *HFE* C282Y variant, which causes the common type of hereditary hemochromatosis, might be involved in determining the variability of iron overload in patients with thalassemia intermedia. Homozygosity for the H63D variant in the *HFE* gene, when coinherited with heterozygous β-thalassemia, seems to increase iron overload. Furthermore, genetic predisposition to osteoporosis (the *VDR*, *COLIAI*, *COLIA2*, and *TGFB1* gene variants) can affect thalassemia trait complications. An increased risk of cardiac complications related to the *GSTM1* haplotype, the *ApoE* ε4 allele, and some HLA haplotypes has been reported in patients with thalassemia major [42,43]. Thus, in the era of molecular medicine, β-thalassemia carriers have a unique opportunity for additional genetic testing and secondary prevention strategy [41,42,44].

Moreover, carrier women of childbearing age should be aware of their diagnosis. During pregnancy, the anemia of thalassemia trait often becomes more severe. Consequently, pregnant women with thalassemia trait would have a higher risk of adverse pregnancy outcomes compared to pregnant women without thalassemia, and a higher level of prenatal care and consultations between obstetricians and hematologists should be considered [45]. Transfusions are rarely necessary, but adequate iron and folate supplementation is recommended to avoid compounding the causes of anemia [28].

## 5. Conclusions

Our results confirm that the accurate diagnosis of heterozygous thalassemia is based on molecular genetic testing. The study identified the spectrum of β-thalassemia variants in Croatia. We believe it is crucial to investigate the population molecular characteristics of thalassemias for effective targeted genetic screening and counselling. More importantly, additional variants in known modifier genes of β-thalassemia should be considered in a follow-up of carriers due to possible secondary complications. Pediatrician’s recommendations for genetic testing and potential special treatment are required. This study provides important new insights into the relevance of β-thalassemia heterozygosity in a pediatric clinical practice in Croatia and globally.

## Figures and Tables

**Table 1:** Frequencies of the *HBB* variants causing β-thalassemia syndromes in Croatia.

Variant	HGVS * Nomenclature	Type of Mutation	β-Thalassemia Carriers	
			*n*	%
Hb Lepore-BW	NG_000007.3:g.63632_71046del	Hb variant	15	32.6
IVSI-110	HBB:c.93-21G>A	β^+^	9	19.6
IVSII-1	HBB:c.315+1G>A	β^0^	6	13.1
IVSI-1	HBB:c.92+1G>A	β^0^	5	10.9
IVSI-6	HBB:c.92+6T>C	β^+^	4	8.7
IVSII-745	HBB:c.316-106C>G	β^+^	2	4.3
Codon 39	HBB:c.118C>T	β^0^	2	4.3
Hb Monroe	HBB:c.92G>C	β^0^	2	4.3
Poly A (A>G)	HBB:c.*111A>G	β^+^	1	2.2
TOTAL			46	100

Hb—hemoglobin; * The HGVS Nomenclature is an internationally recognized standard for describing DNA, RNA, and protein sequence variants.

**Table 2:** Hematological and biochemical features of 46 β-thalassemia carriers.

Hemoglobin [g/L] (*n* = 13), median (range)	113 (91–131)
MCV [fl] (*n* = 15), median (range)	58.4 (51.6–68.5)
MCH [pg] (*n* = 15), median (range)	19.3 (16.5–22)
HbA2 [%] (*n* = 31), median (range)	4.1 (1.8–5.7)
HbF [%] (*n* = 29), median (range)	3.3 (0.4–30.8)

Note: ‘*n*’ represents the total number of carriers for whom the values of the given parameter were available.

**Table 3:** Hematological and biochemical features of β-thalassemia carriers of specific β-globin gene variants.

	Hb Lepore	IVSI-110	IVSII-1	IVSI-1
Hemoglobin [g/L], median (range)	117 (103–131); *n* = 7	117 (113–121); *n* = 2	/	107 (105–109); *n* = 2
MCV [fl], median (range)	61.8 (55.9–64.7); *n* = 6	56.2 (56.1–56.2); *n* = 2	53.6; *n* = 1	58.4 (51.6–59.2); *n* = 3
MCH [pg], median (range)	20.1 (19.3–22); *n* = 6	18.4 (18.1–18.7); *n* = 2	17.1; *n* = 1	19.3 (16.5–19.6); *n* = 3
HbA2 [%], median (range)	2.5 (1.8–2.6); *n* = 11	4.8 (4.1–5.5); *n* = 5	5.4 (5–5.5); *n* = 4	5 (4.3–5.3); *n* = 5
HbF [%], median (range)	3.7 (1.4–30.8); *n* = 10	3.7 (1.7–10.1); *n* = 5	3.3 (1.8–8); *n* = 4	7.7 (1.8–20.9); *n* = 4

Note: ‘*n*’ represents the total number of carriers for whom the values of the given parameter were available.

## Data Availability

The data presented in this study are available on request from the corresponding author. The data are not publicly available due to privacy and ethical restrictions.

## References

[1] Viprakasit V., Ekwattanakit S. (2018). Clinical Classification, Screening and Diagnosis for Thalassemia. Hematol. Oncol. Clin. N. Am..

[2] Mettananda S. (2021). Genetic and Epigenetic Therapies for β-Thalassaemia by Altering the Expression of α-Globin Gene. Front. Genome Ed..

[3] Ali S., Mumtaz S., Shakir H.A., Khan M., Tahir H.M., Mumtaz S., Mughal T.A., Hassan A., Kazmi S.A.R. (2021). Current Status of Beta-thalassemia and Its Treatment Strategies. Mol. Genet. Genomic Med..

[4] Grech L., Borg K., Borg J. (2022). Novel Therapies in β- Thalassemia. Br. J. Clin. Pharmacol..

[5] Kattamis A., Forni G.L., Aydinok Y., Viprakasit V. (2020). Changing Patterns in the Epidemiology of β-Thalassemia. Eur. J. Haematol..

[6] Geography of Croatia—Wikipedia. https://en.wikipedia.org/wiki/Geography_of_Croatia.

[7] Vucak J., Turudic D., Milosevic D., Bilic M., Salek Z., Rincic M., Bilic E. (2018). Genotype-Phenotype Correlation of β-Thalassemia in Croatian Patients: A Specific HBB Gene Mutations. J. Pediatr. Hematol. Oncol..

[8] Efremov G.D. (1992). Hemoglobinopathies in Yugoslavia: An update. Hemoglobin.

[9] Powers J.M., Connor R.F. (2023). Approach to the child with anemia. UpToDate.

[10] Mosca A., Paleari R., Ivaldi G., Galanello R., Giordano P.C. (2009). The role of haemoglobin A(2) testing in the diagnosis of thalassaemias and related haemoglobinopathies. J. Clin. Pathol..

[11] Urosevic J., Djurinovic T., Poznanic J., Cvorkov-Drazic M., Bunjevacki G., Janic D., Krivokapic-Dokmanovic L., Popovic Z., Pavlovic S. (2001). Homogeneity of the Hb Lepore gene in FR Yugoslavia. Balk. J. Med. Genet..

[12] Lamptey H., Seidu Z., Lopez-Perez M., Kyei-Baafour E., Hviid L., Adjei G.O., Ofori M.F. (2023). Impact of Haemoglobinopathies on Asymptomatic Plasmodium Falciparum Infection and Naturally Acquired Immunity among Children in Northern Ghana. Front. Hematol..

[13] Bardón Cancho E.J., García-Morín M., Beléndez C., Velasco P., Benéitez D., Ruiz-Llobet A., Berrueco R., Argilés B., Cervera Á., Salinas J.A. (2020). Update of the Spanish Registry of Haemoglobinopathies in Children and Adults. Med. Clin. (Barc.).

[14] Al-Hakeim H.K., Abdulla A.K., Almulla A.F., Maes M. (2020). Hereditary Haematologic Disorders in Najaf Province-Iraq. Transfus. Clin. Biol. J. Soc. Francaise Transfus. Sang..

[15] Musallam K.M., Lombard L., Kistler K.D., Arregui M., Gilroy K.S., Chamberlain C., Zagadailov E., Ruiz K., Taher A.T. (2023). Epidemiology of Clinically Significant Forms of Alpha- and Beta-Thalassemia: A Global Map of Evidence and Gaps. Am. J. Hematol..

[16] Bellis G., Parant A. (2022). Beta-thalassemia in Mediterranean countries. Findings and outlook. Investig. Geográficas.

[17] Rao E., Kumar Chandraker S., Misha Singh M., Kumar R. (2024). Global Distribution of β-Thalassemia Mutations: An Update. Gene.

[18] Radmilovic M., Zukic B., Stankovic B., Karan-Djurasevic T., Stojiljkovic M., Spasovski V., Tosic N., Dokmanovic L., Janic D., Pavlovic S. (2010). Thalassemia Syndromes in Serbia: An Update. Hemoglobin.

[19] Pasangna J., George E., Nagaratnam M. (2005). Haemoglobin Lepore in a Malay Family: A Case Report. Malays. J. Pathol..

[20] Pavlović S., Savić A., Stojimirović E. (2006). Talasemijski Sindromi-Molekularna Genetika u Savremenoj Dijagnostici: Molecular Genetics and Modern Diagnostics of Thalassemia Syndromes.

[21] Efremov G.D. (2007). Thalassemias and Other Hemoglobinopathies in the Republic of Macedonia. Hemoglobin.

[22] Cherry L., Calo C., Talmaci R., Perrin P., Gavrila L. (2016). β-Thalassemia Haplotypes in Romania in the Context of Genetic Mixing in the Mediterranean Area. Hemoglobin.

[23] Petkov G.H., Efremov G.D. (2007). Molecular Basis of Beta-Thalassemia and Other Hemoglobinopathies in Bulgaria: An Update. Hemoglobin.

[24] Papachatzopoulou A., Kourakli A., Stavrou E.F., Fragou E., Vantarakis A., Patrinos G.P., Athanassiadou A. (2010). Region-Specific Genetic Heterogeneity of HBB Mutation Distribution in South-Western Greece. Hemoglobin.

[25] Gorello P., Arcioni F., Palmieri A., Barbanera Y., Ceccuzzi L., Adami C., Marchesi M., Angius A., Minelli O., Onorato M. (2016). The Molecular Spectrum of β- and α-Thalassemia Mutations in Non-Endemic Umbria, Central Italy. Hemoglobin.

[26] Ringelhann B., Szelenyi J.G., Horanyi M., Svobodova M., Divoky V., Indrak K., Hollân S., Marosi A., Laub M., Huisman T.H. (1993). Molecular Characterization of Beta-Thalassemia in Hungary. Hum. Genet..

[27] Zahed L. (2001). The Spectrum of Beta-Thalassemia Mutations in the Arab Populations. J. Biomed. Biotechnol..

[28] Rivella S., Giardina J. (2012). Thalassemia Syndromes. Hematology: Basic Principles and Practice.

[29] Verma S., Gupta R., Kudesia M., Mathur A., Krishan G., Singh S. (2014). Coexisting Iron Deficiency Anemia and Beta Thalassemia Trait: Effect of Iron Therapy on Red Cell Parameters and Hemoglobin Subtypes. ISRN Hematol..

[30] Needs T., Gonzalez-Mosquera L.F., Lynch D.T. (2024). Beta Thalassemia. StatPearls.

[31] Herbert L., Muncie J., Campbell J.S. (2009). Alpha and Beta Thalassemia. Am. Fam. Physician.

[32] Yaish H.M. Pediatric Thalassemia. Medscape.

[33] Thilakarathne S., Jayaweera U.-P., Premawardhena A. (2024). Unresolved laboratory issues of the heterozygous state of β-thalassemia: A literature review. Haematologica.

[34] Colaco S., Colah R., Nadkarni A. (2022). Significance of Borderline HbA2 Levels in β Thalassemia Carrier Screening. Sci. Rep..

[35] Kattamis C. (2017). The Normal HbA2 Hematological Phenotype of β-Thalassemia Trait. Problems in Detection and Measures to Improve Sensitivity of Screening Tests. J. Hematol. Transfus..

[36] Lin C.-K., Chen L.-P., Chang H.-L., Sung Y.-C. (2014). Underestimation of the Coexistence of Iron Deficiencies and Thalassemia Minors: A Single Institution Experience in Taiwan. Kaohsiung J. Med. Sci..

[37] Boonrusmee S., Thongkhao A., Wongchanchailert M., Mo-Suwan L., Sangsupawanich P. (2022). Coexisting Iron Deficiency Anemia and Thalassemia Traits in Infants: Implication for an Anemia Screening Program. J. Trop. Pediatr..

[38] Hamoodi Q.R., Al-Ani M.H. (2018). Concomitant Iron Deficiency with B-Thalassaemia Minor in Preschool Children in Erbil City. Adv. Med. J..

[39] Harteveld C.L., Achour A., Arkesteijn S.J.G., Ter Huurne J., Verschuren M., Bhagwandien-Bisoen S., Schaap R., Vijfhuizen L., El Idrissi H., Koopmann T.T. (2022). The Hemoglobinopathies, Molecular Disease Mechanisms and Diagnostics. Int. J. Lab. Hematol..

[40] Thein S.L. (2018). Molecular Basis of β Thalassemia and Potential Therapeutic Targets. Blood Cells. Mol. Dis..

[41] Rujito L., Sasongko T.H. (2018). Genetic Background of β Thalassemia Modifier: Recent Update. J. Biomed. Transl. Res..

[42] Galanello R. (2012). Genetic Modifiers of β-Thalassemia. Hematology Education: The Education Program for the Annual Congress of the European Hematology Association.

[43] Galanello R., Origa R. (2010). Beta-Thalassemia. Orphanet J. Rare Dis..

[44] Tesio N., Bauer D.E. (2023). Molecular Basis and Genetic Modifiers of Thalassemia. Hematol. Oncol. Clin. N. Am..

[45] Ruangvutilert P., Phatihattakorn C., Yaiyiam C., Panchalee T. (2023). Pregnancy Outcomes among Women Affected with Thalassemia Traits. Arch. Gynecol. Obstet..

